# Malaria case-management under artemether-lumefantrine treatment policy in Uganda

**DOI:** 10.1186/1475-2875-7-181

**Published:** 2008-09-19

**Authors:** Dejan Zurovac, James K Tibenderana, Joan Nankabirwa, James Ssekitooleko, Julius N Njogu, John B Rwakimari, Sylvia Meek, Ambrose Talisuna, Robert W Snow

**Affiliations:** 1 Malaria Public Health and Epidemiology Group, KEMRI/Wellcome Trust Research Programme, PO Box 43640, 00100 GPO, Nairobi, Kenya; 2 Centre for Tropical Medicine, Nuffield Department of Clinical Medicine, University of Oxford, CCVTM, Oxford, UK; 3 Center for International Health and Development, Boston University School of Public Health, 85 East Concord Street, 5th Floor, Boston, MA 02118, USA; 4 Malaria Consortium, Africa Regional Office, Sturrock Road, Kampala, Uganda; 5 London School of Hygiene and Tropical Medicine, Keppel Street, WC1E 7HT, UK; 6 Ministry of Health, Uganda Malaria Research Centre, Kampala, Uganda; 7 Ministry of Health, National Malaria Control Programme, Kampala, Uganda; 8 Malaria Consortium, Head Office, Leonard Street, London, UK; 9 Ministry Health, Epidemiological Surveillance Division, PO Box 7272, Kampala, Uganda

## Abstract

**Background:**

Case-management with artemether-lumefantrine (AL) is one of the key strategies to control malaria in many African countries. Yet, the reports on translation of AL implementation activities into clinical practice are scarce. Here the quality of AL case-management is reported from Uganda; approximately one year after AL replaced combination of chloroquine and sulphadoxine-pyrimethamine (CQ+SP) as recommended first line treatment for uncomplicated malaria.

**Methods:**

A cross-sectional survey, using a range of quality of care assessment tools, was undertaken at all government and private-not-for-profit facilities in four Ugandan districts. Main outcome measures were AL prescribing, dispensing and counseling practices in comparison with national guidelines, and factors influencing health workers decision to 1) treat for malaria, and 2) prescribe AL.

**Results:**

195 facilities, 232 health workers and 1,763 outpatient consultations were evaluated. Of 1,200 patients who needed treatment with AL according to guidelines, AL was prescribed for 60%, CQ+SP for 14%, quinine for 4%, CQ for 3%, other antimalarials for 3%, and 16% of patients had no antimalarial drug prescribed. AL was prescribed in the correct dose for 95% of patients. Only three out of seven AL counseling and dispensing tasks were performed for more than 50% of patients. Patients were more likely to be treated for malaria if they presented with main complaint of fever (OR = 5.22; 95% CI: 3.61–7.54) and if they were seen by supervised health workers (OR = 1.63; 95% CI: 1.06–2.50); however less likely if they were treated by more qualified health workers (OR = 0.61; 95% CI: 0.40–0.93) and presented with skin problem (OR = 0.29; 95% CI: 0.15–0.55). AL was more likely prescribed if the appropriate weight-specific AL pack was in stock (OR = 6.15; 95% CI: 3.43–11.05) and when CQ was absent (OR = 2.16; 95% CI: 1.09–4.28). Routine AL implementation activities were not associated with better performance.

**Conclusion:**

Although the use of AL was predominant over non-recommended therapies, the quality of AL case-management at the point of care is not yet optimal. There is an urgent need for innovative quality improvement interventions, which should be rigorously tested. Adequate availability of ACTs at the point of care will, however, ultimately determine the success of any performance interventions and ACT policy transitions.

## Background

Forty-one African countries with endemic *Plasmodium falciparum *malaria have recently changed antimalarial drug policies from ineffective monotherapies to highly efficacious artemisinin-based combination therapy (ACT); and among them, 21 countries selected artemether-lumefantrine (AL) as their first-line ACT for uncomplicated malaria [1]. In Uganda, resistance to chloroquine (CQ) and sulphadoxine-pyrimethamine (SP) monotherapies progressed rapidly during the 1990's [2,3] and the efficacy of the subsequent recommendation of a combination of CQ and SP (CQ+SP) was soon compromised [4,5]. In 2004 the decision was made to abandon CQ+SP in favour of AL, however the policy was subsequently revised to include another ACT combination of artesunate and amodiaquine (AS+AQ) as an alternative treatment when AL is not available [6]. Quinine was recommended as the second-line treatment, SP was reserved only for intermittent preventive treatment in pregnancy, and CQ+SP was no longer recommended for the treatment of malaria.

During 2005 and 2006, the Ugandan Ministry of Health implemented the new treatment policy and this process included four key activities. First, national malaria case-management guidelines were revised to incorporate changes in treatment recommendations [7]. Second, between January and May 2006, all government and private-not-for-profit facilities received AL supplies to ensure adequate stocks prior to delivery of the in-service training at the periphery. AL was delivered in the standard four weight-specific blister packages each containing a different number of the same strength tablets. At all facilities AL was provided free of charge, and by the time of our study, there were no deliveries of AS+AQ to health facilities. Third, in-service training for health workers was conducted to implement new guidelines. The rapid cascade training-programme started in February with a national, two-day workshop for 588 senior district health workers who were responsible for the training of all front-line health workers by the end of May 2006. The training for front-line health workers focused on the management of uncomplicated malaria, was organized over one-day at health facilities, and included lectures without practical case scenarios or clinical practice. No training evaluation, further follow up, or supervisory visits of health workers who attended trainings were undertaken. Finally, wall charts reflecting AL case-management recommendations were developed to serve as job-aids [8]. These charts, together with new guidelines, were delivered to health workers either through district health management teams or during the training sessions.

Effective translation of new guidelines into clinical practice is of critical importance to maximize the potential impact of improved, efficacious therapies. However, there are very few reports on the quality of clinical practices following implementation of AL policies in Africa [9,10]. Here AL case-management practices in accordance with national case-management guidelines are reported approximately 1 year after AL was distributed, training of health providers completed and guidelines and wall charts delivered to peripheral facilities across Uganda.

## Methods

### Study design and data collection

A cross-sectional, cluster sample survey was conducted between 29 May and 16 August 2007 at all government and private-not-for profit health facilities in four districts purposively selected to represent diversity of malaria transmission in Uganda: Apac, rural holoendemic district; Tororo, rural hyperendemic district; Mubende, rural mesoendemic district; and Jinja, periurban mesoendemic district [11]. A cluster was defined as all consultations for sick patients presenting at each facility on a randomly selected, single survey day. Patients coming for follow up visits for chronic diseases (e.g. TB, diabetes), traumas, burns, and patients referred or admitted for hospitalization were not recruited.

Sixteen nurses in eight survey teams collected data. In each district training and concordance testing was undertaken over five days until the agreement of practice results of nurses and trainers was greater than 90%. At health facilities data were collected using a range of quality of care assessment methods. All adult patients and caretakers of sick children were interviewed and had limited clinical screening when they completed their health facility visit and were ready to leave. Prior to the interviews, written informed consent to be enrolled in the study was obtained from all patients or their caretakers. Interviewers determined the patient's age, weight, history of fever, axillary temperature, main complaints, prior use of antimalarial drugs, routine counseling and drug dispensing practices undertaken during the facility visit, and if the visit was an initial or follow-up consultation. Information was also collected from patient-held records about routine diagnostic procedures requested, results reported and medications prescribed.

At the end of the survey day all health workers who had attended study patients were interviewed to collect information on their demographics, pre-service and in-service training, working experience, access to national guidelines and exposure to supervision in the preceding six months. Finally, a health facility assessment was undertaken to record the availability of AL and other antimalarials on the survey day and in the past 6 months, the presence of functional weighing scales, thermometers, malaria diagnostics, and any displayed case-management wall charts. In addition, malaria treatments prescribed during the week prior to the survey were documented from the patients' registers held at the clinic.

### Definitions

Study definitions reflected national malaria case-management recommendations from the latest Ugandan *"Guideline for Health Workers on the Management of Uncomplicated Malaria*" [7], "*Guidelines for Integrated Management of Childhood Illnesses*" [12] and "*Flowcharts for Management of Malaria*" [8]. In summary, according to these guidelines and flowcharts any patient presenting with fever or history of fever in absence of danger signs and prior, correct use of ACTs should be presumptively treated with recommended first line treatment for uncomplicated malaria. The guidelines provide the same recommendations across all age groups. The use of malaria microscopy is discouraged for most febrile patients with the exception of special patient groups such as suspected treatment failures, severe cases, children below 5 kg and pregnant women. A case of patient needing management with AL was defined as a non-pregnant patient, weighing 5 kg and above, who presented to a health facility for an initial visit with a history of fever during the present illness or axillary temperature ≥ 37.5°C, and treated as an outpatient in the absence of a negative routine malaria test and prior use of ACT.

The correctness of AL dosage prescriptions was assessed in accordance with guidelines dosage recommendations for four weight-specific AL categories [7] and was classified into three categories: 1) recommended (one tablet twice a day over three days for a 5–14 kg patient; two tablets twice a day over three days for a 15–24 kg patient; three tablets twice a day over three days for a 25–34 kg patient; and four tablets twice a day over three days for a patient 35 kg and above, 2) overdosed, and 3) underdosed prescriptions. The quality of AL dispensing and counseling for patients included the performance of the following seven tasks: weighing of patients, explanation on how to take AL at home, administration of the first dose at health facility, observation of the swallowing of the first dose, and provision of advises to take AL after the meal, to complete all doses, and what to do in case of vomiting.

### Statistical analysis

Data were double-entered into Microsoft Access 2000 (Microsoft Inc, Redmond, Washington). Questionnaires were entered twice by two independent data entry clerks, and data files were compared for errors using a verification programme and referring to original questionnaires. All analyses were performed using STATA, version 8 (StataCorp, College Station, Texas). Results from four districts were combined and descriptive analysis was undertaken at the health facility, health worker, and patient level across all age groups and stratified for children below 5 years of age and patients 5 years and older. A stepwise approach was applied in analyzing quality of malaria case-management for patients who needed to have AL prescribed according to guidelines. First, to assess overall performance of the new policy, treatment practices were analyzed at all health facilities regardless of the availability of AL. Then, to assess health workers adherence to the new policy analysis was restricted to facilities where AL was in stock on the day of the survey. Finally, at facilities with available AL, the quality of AL dosage prescriptions, and the quality of dispensing and counseling practices was respectively restricted to patients who had AL prescribed and to those who had both, AL prescribed and dispensed at facility. Data are presented as frequencies and proportions, with corresponding 95% confidence intervals (CIs) adjusted for clustering by health facility.

Two outcomes were selected to examine factors influencing health workers adherence to guidelines at facilities where AL was in stock. These included health workers decision to 1) treat for malaria, and 2) select AL as the recommended drug. The following factors were examined: health workers pre-service training; in-service training on the use of AL; access to national malaria guidelines; presence of AL wall charts; availability of CQ; availability of weight-specific AL packs; supervision including AL (defined as at least one supervisory visit in past 6 months including any discussion on appropriate use of AL); the patient's age; and main complaints of fever, cough, and skin problem. Interaction terms between in-service training and all factors of programmatic importance were also tested for each of two outcomes. The association between factors and outcomes was examined applying logistic regression modeling with the STATA *xtgee *procedure using an exchangeable working correlation matrix to account for correlated nature of data [13]. For each outcome the odds ratio (OR), 95% confidence interval and *P*-values were first estimated for each factor in a series of univariate models. All factors with *P*-value for association < 0.15 were then entered into multivariate model, and a backward, stepwise strategy was used to eliminate variables with *P*-value > 0.05. Hypothesis testing and confidence interval estimation were done with an alpha level of 0.05.

### Ethical approval

The ethical approval for this study was provided by the Uganda National Council for Science and Technology (reference number HS 275).

## Results

### Description of the sample

All 195 health facilities which were open during the survey period in study districts were assessed. At seven facilities, a health facility assessment was undertaken but no consultations took place during the survey day. At the remaining 188 facilities, outpatient malaria case-management practices were evaluated on 1,766 consultations undertaken by 233 health workers. No health worker, adult patient or caretaker on behalf of sick child refused to participate in the study. However, of 233 health workers, one (0.4%), who performed three consultations, had to urgently leave the facility before an interview could be performed. Of the 1,763 consultations analyzed, 1,200 consultations met our definition of patients who needed AL management in accordance with national guidelines (461 < 5 years and 739 ≥ 5 years of age). The remaining 563 consultations were for patients < 5 kg (10), pregnant women (74), follow up visits (94), had neither a history of fever nor temperature ≥ 37.5°C (371), had received AL prior to coming to facility (47), or had negative malaria slide reported through routine practices (85).

### Health facility and health worker characteristics

Of the 195 health facilities assessed, most (90%) were smaller health centres II and III, and most facilities were run by the government (88%) (Table 1). The majority had functional weighing scales (91%) and thermometers (75%) while malaria microscopy was provided at only 26% of facilities. Only one facility stocked malaria rapid diagnostic tests. Nearly half of facilities (93/195; 48%) had displayed at least one wall chart recommending use of AL, most commonly the chart on management of uncomplicated malaria (68/93; 73%). Only 17% of facilities had IMCI charts revised to recommend AL use.

**Table 1:** Characteristics of the health facilities in study districts in Uganda

**Characteristics (N = 195)**	**n (%)**
Health facility type	
Hospital	8 (4.1)
Health centre IV	12 (6.2)
Health centre III	56 (28.7)
Health centre II*	119 (61.0)
Health facility ownership	
Government	172 (88.2)
Non-government	12 (6.2)
Mission	11 (5.6)
Equipment and services at health facility	
Weighing scale	178 (91.3)
Thermometer	146 (74.9)
Functional microscopy	50 (25.6)
Wall charts exposed	
Any chart recommending artemether-lumefantrine use	93 (47.7)
Chart on uncomplicated malaria	68 (34.9)
Chart on new treatment policy	35 (18.0)
Integrated management of childhood illnesses chart	33 (16.9)
Availability of artemether-lumefantrine on the survey day	
Any tablets of artemether-lumefantrine	169 (86.7)
Artemether-lumefantrine 6 tablets pack	162 (83.1)
Artemether-lumefantrine 12 tablets pack	140 (71.8)
Artemether-lumefantrine 18 tablets pack	75 (38.5)
Artemether-lumefantrine 24 tablets pack	124 (63.6)
Availability of other antimalarial drugs on the survey day^†^	
Chloroquine (any formulation)	149 (76.8)
Sulphadoxine-pyrimethamine (any formulation)	170 (87.6)
Quinine (tablets)	58 (29.9)
Amodiaquine (any formulation)	4 (2.1)
Artesunate tablets	8 (4.1)

* This category includes 3 clinics of similar service capacity as health centre II

† Denominators for these variables do not include one health facility with missing value

On the survey day, any tablet packs of AL were in stock at 87% of facilities, the availability ranging from 39% for 18 tablet packs to 83% for 6 tablet packs (Table 1). All four weight-specific AL packs were available at only 67 (34%) facilities. Other antimalarial drugs recommended for treatment of uncomplicated malaria such as quinine, artesunate and amodiaquine were respectively in stock at only 30%, 4%, and 2% of facilities. Conversely, non-recommended antimalarials were widely available; CQ at 77% and SP at 88% of facilities. Of 157 facilities where retrospective stock-out data were available, the stock-outs of AL during the six-month period prior to the survey were common: 75% of facilities experienced stock-out of at least one of four AL tablet packs; between 44% and 68% reported stock-outs for specific AL packs; and 35% of facilities were found to have simultaneous stock-out of all four packs. In these facilities, the median number of the stock-out days without any AL was 60 [IQR: 27–113] or 33% of study time. During the same retrospective period, CQ was rarely out of stock (23%), while most facilities reported stock-outs of quinine (58%), artesunate (98%) and amodiaquine (98%).

Of 232 interviewed health workers, the most common cadre performing consultations were nurses (35%); 24% were clinical officers and doctors; 4% midwives; and further 38% of health workers were health workers without formal clinical training, most commonly nurse aids and nursing assistants (Table 2). The majority of health workers (182/232; 79%) were trained on AL use, most commonly through the MoH cascade in-service training programme (124/182; 68%). The coverage of health workers trained on IMCI was high (160/232; 69%), however only 9 of these health workers attended recent IMCI trainings that included AL recommendations. Overall, higher cadres of health workers were more likely to have been exposed to AL training (86% of clinical officers and doctors vs 82% of nurses and midwives vs 71% of those without formal clinical training). 68% of health workers had access to national AL guidelines and 54% had access to IMCI chart booklets which were, however, not revised to include AL treatment recommendations. More than half (52%) of health workers reported at least one supervisory visit in past six months that included discussion on any clinical topic, however only 34% health workers reported a visit that included topic on appropriate use of AL.

**Table 2:** Characteristics of the health workers in study districts in Uganda

**Characteristics (N = 232)**	**n (%)**
Pre-service training	
Doctor	3 (1.3)
Clinical officer	52 (22.4)
Nurse	80 (34.5)
Midwife	9 (3.9)
Cadre without formal clinical training*	88 (37.9)
In-service training on malaria	
Any training including artemether-lumefantrine	182 (78.5)
MoH training on artemether-lumefantrine	124 (53.5)
On-job training on artemether-lumefantrine	107 (46.1)
IMCI training including artemether-lumefantrine^†^	9 (3.9)
Any IMCI training	160 (69.0)
Possession of guideline document	
Any guideline including artemether-lumefantrine	158 (68.1)
Management of uncomplicated malaria	153 (66.0)
New policy for uncomplicated malaria	58 (25.0)
Any supervisory visit including appropriate use of AL^†^	78 (33.8)

*This category includes 25 nurse aids, 53 nurse assistants, 3 TB/leprosy assistant, 3 vaccinators, 1PH dental officer, 1 entomologic assistant, 1 anesthetic officer and 1 laboratory technician

^† ^Denominators for these variables do not include one health worker with missing value

### Quality of AL prescribing, dispensing and counseling practices

Of 1,200 patients weighing 5 kg and above who needed treatment with AL according to guidelines and presenting to all study facilities, AL was prescribed for 60% of patients, CQ+SP for 14%, quinine for 4%, CQ for 3%, and various other antimalarials for 3% of patients (Table 3). Notably, among all patients 16% left the facility with no antimalarial prescription, similar in children < 5 years (14%) compared to patients 5 years and older (17%). At the same facilities, the retrospective one-week review of 18,917 antimalarial prescriptions adjusted for the same rate of non-treated febrile patients as observed on the survey day (16%), suggested somewhat lower use of AL (52%), similar use of CQ+SP (13%), and more common prescriptions of quinine (7%), CQ (7%) and SP (4%).

**Table 3:** Antimalarial treatments for patients who needed management with AL as defined by national guidelines

***All health facilities***	**< 5 years (N = 461)**		**≥ 5 years (N = 739)**		**All patients (N = 1200)**	
	
	**n (%)**	**95% CI**	**n (%)**	**95% CI**	**n (%)**	**95% CI**
AL	306 (66.4)	61.1–71.7	415 (56.2)	50.3–62.0	721 (60.1)	55.6–64.6
CQ+SP	35 (7.6)	4.8–10.4	133 (18.0)	13.2–22.8	168 (14.0)	10.7–17.3
Quinine	27 (5.9)	3.1–8.6	21 (2.8)	1.4–4.3	48 (4.0)	2.6–5.4
Chloroquine	10 (2.2)	0.7–3.6	22 (3.0)	1.4–4.6	32 (2.7)	1.5–3.9
SP	2 (0.4)	0–1.0	7 (1.0)	0–2.1	9 (0.8)	0–1.5
Other antimalarial treatments*	16 (3.5)	1.4–5.6	14 (1.9)	0.5–3.3	30 (2.5)	1.2–3.8
No antimalarial prescribed	65 (14.1)	10.8–17.4	127 (17.2)	13.9–20.5	192 (16.0)	13.5–18.5

***Facilities with AL in stock***	**< 5 years (N = 428)**		**≥ 5 years (N = 644)**		**All patients (N = 1072)**	
	
	**n (%)**	**95% CI**	**n (%)**	**95% CI**	**n (%)**	**95% CI**

AL	297 (69.4)	64.2–74.6	388 (60.3)	54.2–66.3	685 (63.9)	59.5–68.3
CQ+SP	22 (5.1)	2.8–7.5	95 (14.8)	10.1–19.4	117 (10.9)	8.0–13.9
Quinine	22 (5.1)	2.4–7.9	16 (2.5)	1.0–4.0	38 (3.5)	2.2–4.9
Chloroquine	9 (2.1)	0.6–3.6	16 (2.5)	0.8–4.2	25(2.3)	1.1–3.6
SP	2 (0.5)	0–1.1	6 (0.9)	0–2.2	8 (0.8)	0–1.6
Other antimalarial treatments^†^	15 (3.5)	1.3–5.7	12 (1.9)	0.3–3.4	27 (2.5)	1.1–4.0
No antimalarial prescribed	61 (14.3)	10.8–17.7	111 (17.2)	13.8–20.7	172 (16.0)	13.4–18.7

* Other antimalarial treatments include artemether (2), AQ+SP (2), AL+SP (8), AL+CQ+SP (2), QN+SP (6), AL+QN (4), AL+CQ (6)

† Other antimalarial treatments include artemether (1), AQ+SP (2), AL+SP (8), AL+CQ+SP (2), QN+SP (6), AL+QN (3), AL+CQ (5)

Surprisingly, at facilities where AL was in stock on the day of the survey, only minor and statistically non-significant changes in health workers adherence to the new policy were observed compared to practices at all facilities: AL prescribing increased by 4% (60 to 64%), use of CQ+SP decreased by 3% (14 to 11%), other antimalarials for only 1% (10 to 9%), and the proportion of patients not treated with any antimalarial drugs remained exactly the same (16%) (Table 3).

Among 714 patients who had AL prescribed and for whom dosing prescriptions were complete (7 missing values), 676 (95%) were prescribed recommended AL weight-specific dosages while overdose and underdose prescriptions were very rare (16/714; 2% and 22/714; 3% respectively). High rates of recommended dosage prescriptions were observed across all weight groups: 5–14 kg (273/283; 97%), 15–24 kg (79/89; 89%), 25–34 kg (10/15; 67%) and 35 kg and above (314/325; 97%). Conversely, recommended CQ and quinine dosage prescriptions were less common (17/32; 53% and 27/48; 56%).

For 669 patients who had AL dispensed at the health facility (Table 4), health workers variably performed dispensing and counseling tasks: 51% had weight measured, 97% were explained how to take AL at home, 67% were advised to complete all doses, 47% were instructed to take drug after the meal, 15% received the first dose of AL while at the facility, 14% were observed while swallowing the first dose, and only 7% were advised what do in case of vomiting. Interestingly, apart from measuring the weight which health workers more commonly performed for children < 5 years of age (65%) than in patients 5 years and older (41%), there was no significant difference between age groups in the performance of any other dispensing and counseling tasks (Table 4).

**Table 4:** Quality of dispensing and counseling practices for patients who had AL dispensed

***Task performed***	**< 5 years (N = 296)**		**≥ 5 years (N = 373)**		**All patients (N = 669)**	
	
	**n (%)**	**95% CI**	**n (%)**	**95% CI**	**n (%)**	**95% CI**
Weight measured	192 (64.9)	55.9–73.8	151 (40.5)	30.9–50.1	343 (51.3)	43.2–59.4
First dose given at the facility	40 (13.5)	7.4–19.7	58 (15.6)	8.4–22.7	98 (14.7)	8.5–20.8
Swallowing of first dose observed	34 (11.5)	5.3–17.6	56 (15.0)	7.8–22.2	90(13.5)	7.3–19.7
Dosage explained	284 (96.0)	93.3–98.6	363 (97.3)	95.1–99.5	647 (96.7)	94.7–98.7
Advice provided to complete all doses	185 (62.5)	55.1–69.9	262 (70.2)	64.1–76.4	447 (66.8)	61.3–72.3
Advice provided to take drug after a meal	132 (44.6)	37.1–52.1	182 (48.8)	42.7–54.9	314 (46.9)	41.3–52.6
Advice provided what to do if vomiting	24 (8.1)	2.9–13.3	20 (5.4)	2.1–8.6	44 (6.6)	3.1–10.1

### Factors influencing quality of AL case-management

Eleven factors and six interaction terms that may have influenced health workers decisions to 1) treat for malaria, and 2) select recommended AL treatment at facilities where AL was in stock on the survey day were examined. Table 5 presents multivariate results for statistically significant associations (*P*-value < 0.05) between examined factors and each outcome, and univariate results for factors which did not meet the criteria for multivariate analysis (*P*-value < 0.15) or were statistically non-significant to be retained in final models.

**Table 5:** Factors influencing health worker's practices in management of patients with artemether-lumefantrine

Outcomes and factors (No of patients in analysis*)	No (%) of patients with outcome	% of patients with the outcome for each level of the factor	OR (95% CI)	*P-* value
**Statistically significant factors (P < 0.05): multivariate results**				

**Outcome 1: Health worker treats for malaria (n = 1064)**	892 (83.8)			
Fever main complaint (yes vs no)		88.6 vs 60.6	5.22 (3.61–7.54)	< 0.001
Supervision including AL (supervised vs non-supervised HWs)		87.2 vs 82.1	1.63 (1.06–2.50)	0.027
Health worker's cadre (qualified^† ^vs non-qualified)		82.1 vs 86.7	0.61 (0.40–0.93)	0.020
Skin problem main complaint (yes vs no)		65.3 vs 84.7	0.29 (0.15–0.55)	< 0.001
				
**Outcome 2: Health worker selects AL treatment (n = 892)**	682 (76.5)			
Availability of weight-specific AL pack (yes vs no)		83.8 vs 43.6	6.15 (3.43–11.05)	< 0.001
Absence of chloroquine in stock (yes vs no)		84.3 vs 74.1	2.16 (1.09–4.28)	0.027

**Statistically non-significant factors – univariate results**				

**Outcome 1: Health worker treats for malaria (n = 1064)**	892 (83.8)			
Malaria in-service training including AL (yes vs no)		82.8 vs 87.9	0.65 (0.41–1.05)	0.078
Access to malaria guidelines (yes vs no)		84.8 vs 81.8	1.19 (0.81–1.76)	0.370
Availability of malaria wall charts (yes vs no)		83.8 vs 83.9	1.02 (0.70–1.50)	0.906
Availability of weight-specific AL pack (yes vs no)		83.5 vs 85.3	0.87 (0.49–1.55)	0.635
Absence of chloroquine in stock (yes vs no)		84.7 vs 83.6	1.06 (0.66–1.70)	0.819
Age of the patient (<5 years vs ≥ 5 years)		85.7 vs 82.6	1.27 (0.88–1.82)	0.203
Cough main complaint (yes vs no)		84.6 vs 83.0	1.09 (0.78–1.53)	0.598
				
**Outcome 2: Health worker selects AL treatment (n = 892)**	682 (76.5)			
Malaria in-service training including AL (yes vs no)		77.4 vs 73.0	1.37 (0.78–2.40)	0.267
Access to malaria guidelines (yes vs no)		76.8 vs 75.6	1.12 (0.68–1.86)	0.656
Availability of malaria wall charts (yes vs no)		76.5 vs 76.4	0.93 (0.58–1.50)	0.778
Health worker's cadre (qualified^† ^vs non-qualified)		74.8 vs 79.0	0.88 (0.55–1.41)	0.583
Supervision including AL (supervised vs non-supervised HWs)		79.6 vs 74.7	1.41 (0.85–2.32)	0.180
Age of the patient (< 5 years vs = 5 years)		81.2 vs 73.2	1.59 (1.02–2.47)	0.039
Fever main complaint (yes vs no)		75.9 vs 80.7	0.81 (0.52–1.26)	0.345
Cough main complaint (yes vs no)		79.2 vs 73.5	1.32 (0.98–1.77)	0.064
Skin problem main complaint (yes vs no)		78.1 vs 76.4	1.03 (0.43–2.47)	0.943

* 8 observations with incomplete data omitted to allow for a common denominator

† The category qualified heath workers include patients seen by doctors, nurses, clinical officers and midwives.

Of 1,064 patients who should have been treated for malaria in accordance with guidelines, multivariate results revealed significantly higher likelihood of recommended practice for patients with main complaint of fever (OR = 5.22; 95% CI: 3.61–7.54) and for those patients seen by supervised health workers (OR = 1.63; 95% CI: 1.06–2.50). Patients were significantly less likely to be treated for malaria if they were seen by formally qualified health workers (OR = 0.61; 95% CI: 0.40–0.93) or if they presented with a skin problem (OR = 0.29; 95% CI: 0.15–0.55). Among 892 patients who were prescribed an antimalarial drug, AL was significantly more likely to be prescribed if they were seen at facilities where on the survey day the appropriate weight-specific AL pack was in stock (OR = 6.15; 95% CI: 3.43–11.05) and where CQ was absent (OR = 2.16; 95% CI: 1.09–4.28). Interestingly, no significant association was found between any of AL implementation activities (in-service training including AL, access to national guidelines, and availability of malaria wall charts) and tested outcomes (Table 5). Similarly, no significant association was found with any of outcomes for the effects of in-service training in interaction terms with guidelines, wall charts, supervision, pre-service training, availability of weight-specific AL, and absence of CQ.

## Discussion

This study of AL case-management was undertaken approximately three years after the policy shift from CQ+SP to AL was announced in Uganda, two years after the beginning of implementation activities and at least a year after AL was delivered to all facilities in study districts. This provided sufficient time for health workers to become familiarized with the new guidelines and translate new policy effectively into the clinical practice.

Despite the lack of universal availability of AL on the survey day (87%) and less than optimal coverage with in-service training (79%), guidelines (68%) and AL wall charts (48%), the overall use of AL prevailed (60%) over non-effective CQ, SP and CQ+SP therapies (18%), or effective, but not recommended therapies, such as quinine and other antimalarial combinations (7%). The presence of study teams at facilities may have introduced a Hawthorn effect resulting in better performance on the survey day than usual [14,15]; however, the retrospective review of treatment practices in a week prior to the presence of study teams suggests that this effect was relatively minor as 52% of patients were still prescribed AL prior to the survey. Unexpectedly, no further improvements were observed at facilities where AL was in stock on the survey day. At these facilities two main practices discordant with national recommendations are important to emphasize: first, 20% of patients were still prescribed non-recommended antimalarials, most commonly CQ+SP, and second, further 16% of febrile patients were not treated at all for malaria. Our explanatory analyses provide some explanations that help to understand these patterns.

First, health workers were much more likely to prescribe AL if weight-specific AL pack was in stock (OR = 6.2), but also if non-recommended chloroquine was absent (OR = 2.2). The negative effects of the presence of ineffective drugs on treatment practices have been described in Kenya [16,17], and the implications of these findings are clear – discontinuation of supply and removal of existing stocks from facilities. More than a year after the new policy reached peripheral facilities, the absence of a stable AL supply chain does not justify the continued supply of ineffective drugs.

Controversy, however, exists on the most appropriate ways to approach common situations where tablets of AL are in stock but not in adequate pack sizes. The strength of all AL tablets is indeed the same, however the implementation of the AL policy includes delivery of four different AL pack sizes (6, 12, 18 and 24 tablets) suitable for management of four different weight categories of patients (5–14 kg; 15–24 kg; 25–34 kg and ≥ 35 kg). In Uganda there are no instructions on what health workers should do when only non-recommended weight-specific AL is in stock. An ideal solution to this problem is establishment of an effective supply chain for all four AL products; however this seems to have taken longer than anticipated. Meanwhile, the findings of this study support recommendations to instruct health workers to use AL even if adequate AL pack sizes are not in stock. This temporary practice may compromise high levels of patients' adherence to AL [18], and high rates (95%) of health workers adherence to correct AL dosing as shown in this and in the previous studies [9,10]; however, it would still save many lives before an effective AL supply chain is established countrywide. If adequate availability of AL products cannot be ensured, alternative AL preparations that do not depend on separate packaging, should be considered and their operational use evaluated. Finally, the implementation of parallel ACT policy promoting AS+AQ, as a stipulated recommendation in national guidelines in Uganda, might also be an option to support sufficient availability of ACTs at the periphery of health system.

The second practice discordant with guidelines is that 16% of febrile patients are not treated with any antimalarial drug; the proportion similar between young children and patients 5 years and older. Health workers are more likely to respect guidelines if febrile patients spontaneously report fever, or present without additional problems such as skin infection. Health workers with higher qualifications and those less supervised are more likely to disregard guidelines. These findings are similar to those reported previously in Benin [19] and Kenya [16] and continue to persist despite many studies in the past demonstrating low sensitivity of various clinical signs and symptoms to detect malaria in febrile patients across all age groups [20-22], translation of this evidence into national [7,12] and international guidelines [23,24], and emphasis of series of Ugandan in-service training programmes on these guidelines [25]. This practice is more prominent among more qualified health workers and possibly reflects the common beliefs that their skills, and knowledge of alternative diagnoses, could reliably distinguish malaria fevers from other febrile causes. The advent of malaria rapid diagnostic tests may present an opportunity not only to address well-known problem of malaria overdiagnosis [26], but also to reduce the risk of malaria under-treatment by promoting systematic testing of all febrile patients.

Appropriate AL dispensing and counseling practices deserve special attention. The performance of these tasks, as they should be provided as part of good clinical practice, are critical to ensure high rates of patients adherence [18] and treatment success [27,28]. In this study, we found that nearly all patients left the facility with an explanation on dosing schedule; however, administration of the first AL dose, and provision of advice to take AL after the meal and what to do in case of vomiting was rarely performed across all age groups. Despite Uganda's investment in interventions to improve the quality of care such as IMCI [25,29], deficiencies in drug dispensing and counseling practices persist. The reasons for these suboptimal practices are not clear and demand further qualitative research including health workers misperceptions on administration of first AL dose in the absence of food, the effects of lack of potable water on administering drugs at peripheral facilities and the effects of AL blister packages on the provision of replacement dose in case of vomiting. Better understanding of these factors should guide further interventional studies to improve dispensing and counseling practices as an integral part of appropriate prescribing.

Finally, better prescribing practices could not be attributed to any routine AL implementation activities (in-service training, guidelines, wall charts). The reviews of previous studies with similar design, as well as systematic reviews of other interventional trials on the use of medicines in developing countries, commonly reported a mixed association between in-service training and health worker performance, and sometimes, as in the present study, no association was found with any of the examined interventions [30-32]. Unfortunately, most studies, including this one, had only limited details about the quality of training delivered to health workers in study settings to make any definite conclusions about effectiveness of this intervention. Yet, it seems obvious that currently established ways of delivering ACT policies and ensuring health workers performance during the post-delivery period need strengthening. Prospective, interventional studies testing cost-effectiveness of innovative types of training, but also job-aids and supervision, and their combinations, should be an operational research priority of critical importance to inform policy makers on how to optimize the delivery of ACTs in Africa.

## Conclusion

Although it was encouraging to observe that the use of AL prevailed over non-recommended therapies in Uganda, the quality of AL case-management is not yet optimal and a series of deficiencies were detected at point of care. Following widespread ACT deployment across Africa, the priority for operational researchers and policy makers should be to determine the most cost-effective set of interventions that can be easily deployed on a larger scale to maximize the impact of ACTs so long as their adequate supply to the periphery of health system can be guaranteed.

## Competing interests

DZ, AT and RWS have received a fee for speaking at a meeting organized by Novartis Pharma AG, the manufacturers of artemether-lumefantrine. JKT, JN, JS, JNN, SM and JBR declared no competing interest.

## Authors' contributions

DZ contributed to the conception and design of the study, analysis, interpretation of results and finalization of the manuscript. JKT contributed to the conception and design of the study, supervision of data collection and interpretation of results. JN contributed to study design, supervision of data collection and data analysis. JS and JNN contributed to data analysis and interpretation of results. JBR contributed to data analysis, interpretation of results and policy implications of the findings. AT contributed to the conception and design of the study. SM contributed to interpretation of results and policy implications of the findings. RWS contributed to the conception and design of the study and interpretation of results. All authors contributed to drafting of the manuscript and all read and approved the final manuscript.
